# Regenerative Efficacy of Supercritical Carbon Dioxide-Derived Bone Graft Putty in Rabbit Bone Defect Model

**DOI:** 10.3390/biomedicines10112802

**Published:** 2022-11-03

**Authors:** Yen-Lung Chiu, Yun-Li Luo, Yuan-Wu Chen, Chi-Tsung Wu, Srinivasan Periasamy, Ko-Chung Yen, Dar-Jen Hsieh

**Affiliations:** 1 Department of Life Sciences, National Cheng Kung University, Tainan 70101, Taiwan; 2 Division of Oral and Maxillofacial Surgery, Tri-Service General Hospital, Taipei City 110, Taiwan; 3 School of Dentistry, National Defense Medical Center, Taipei City 11490, Taiwan; 4 R&D Center, ACRO Biomedical Co., Ltd., Kaohsiung 82151, Taiwan

**Keywords:** porcine bone graft putty, supercritical carbon dioxide, bone regeneration, critical bone defect rabbit model

## Abstract

Bone defects can arise from numerous reasons, such as aging, tumor, trauma, infection, surgery, and congenital diseases. Bone grafts are commonly used as a substitute to fill the void and regenerate the defect. Due to its clean and green technology, the supercritical carbon dioxide (SCCO_2_) extraction aided the production of bone grafts is a recent trend. The SCCO_2_-derived bone graft has osteoconductive and osteoinductive properties along with excellent biocompatible, nontoxic, bioabsorbable, osteoconductive, and good mechanical properties; however, clinical usage during surgery is time-consuming. Therefore, we produced a putty material combining bone graft powder and acellular dermal matrix (ADM) powder and tested its regenerative efficacy in the critical defect in the rabbit model. The putty was found to retain the tubular structure. In addition, the putty depicted excellent stickiness and cohesiveness in both saline and blood medium. The bone regeneration of bone graft and putty was similar; both had excellent bone healing and regeneration of critical defects as evaluated by the X-ray, microtomography, hematoxylin-eosin, Masson trichrome, and alizarin red staining. Putty contains a less washout rate, good mechanical strength, and biocompatibility. In conclusion, the SCCO_2_-derived moldable putty could be a promising easy-to-use alternative for bone grafts at present which might have real-world usage in orthopedics as a potential bone void filler and dental socket preservation.

## 1. Introduction

Aging, tumors, trauma, infection, and surgery are the various causes that lead to bone defects [1,2]. To treat the bone defect, the most frequently used approach is bone grafting, in which bone grafts are extensively used for bone regeneration in orthopedic surgeries [3]. The bone graft matrices aim to encourage bone tissue regeneration by degrading itself and substituting it with freshly created bone at the defect site. Conventional bone grafts are produced to substitute the missing bone tissue by filling the defect and alleviating the bone damage [4]. The perfect bone graft essentially enhances bone healing and regeneration with ideal steadiness and robustness, along with features including osteogenesis, osteoinduction, and osteoconduction [2,5]. 

At present, bone grafts are aimed at functionally regenerating the bone tissue using bioactive materials [4]. Regardless of the developments towards this goal, until now, there has been no bone graft material to substitute autogenous bone that owns all the necessary characteristics, including osteoinductivity, biological safety, a long shelf life, and cost-effectiveness [6]. At present autologous bone graft is the sole biomaterial that accomplishes the above-mentioned standards and signifies the “gold standard” graft for bone defect repair without graft rejection. However, the scarcity of supply, donor site injury, lengthy hospital stay, and, most importantly, the increased risk of surgical site infections lead to limited common use in clinical practice. This subsequently provoked the researchers to discover possible substitutes such as synthetic, allograft, and xenograft bone substitutes. These bone alternatives have some rewards, such as no shortage in the supply because of accessibility of donors, including allo and xenografts, while the synthetic bone alternates can be produced with anticipated quantity and quality [2,7].

Bone grafts can be from animal sources; these xenogenic bone grafts are from bovine, ovine, equine, or porcine sources and are available commercially. The existing standard procedure for manufacturing those xenogenic bone grafts is done by high-temperature sintering at 300–1300 °C. This procedure permits the overall eradication of any likely zoonotic infectious agents and immunogenic components in the xenogenic bone. However, the high temperature also breaks down and eliminates the intrinsic collagen and changes the porous structures of the xenogenic bones that unavoidably disrupt the extracellular matrix (ECM) structure, [8] which can trigger adverse reactions that lead to a rise in bone resorption and encapsulating fibrosis [9]. However, these drawbacks can be overcome by producing bone grafts employing a supercritical carbon dioxide (SCCO_2_) extraction technology [10].

The production of bone grafts by SCCO_2_ extraction technology uses supercritical fluid CO_2_ as the extracting solvent to remove the fats, cells, and non-collagenous proteins from the xenogenic bone and other tissues. The advantages of the SCCO_2_ extraction technology are as follows: it is natural, safe, non-toxic, non-corrosive, non-flammable, easily accessible, and cost-effective. The SCCO_2_ extraction technology employs mild critical coordinates, such as pressure at 7.38 MPa and temperature at 31 °C, which can be easily attained and are well-matched for biological materials. We have successfully produced bone grafts and an acellular dermal matrix using SCCO_2_ extraction technology [10,11].

We have produced bone grafts from porcine bone and acellular dermal matrix from porcine hide using SCCO_2_ technology to manufacture bone matrix branded as ABCcolla^®^ Collagen Bone Graft and ABCcolla^®^ Collagen Matrix (ACRO Biomedical Co., Ltd. Kaohsiung City, Taiwan). Both are certified by U.S. Food and Drug Administration (FDA) and Taiwan Food and Drug Administration (TFDA). The present study aims to formulate a bone graft putty by combining bone matrix powder and acellular dermal matrix powder and evaluating the putty’s physical nature and usability. In addition, we evaluated the bone regenerative efficacy of the putty by the X-ray, microtomography, hematoxylin-eosin, Masson trichrome, and alizarin red staining in the rabbit femoral defect model as the main objective in this study.

## 2. Materials and Methods

### 2.1. Development of Bone Graft

Bone graft was produced, as explained by Chen et al. (2021) [10]. In brief, the porcine bone was procured from Tissue Source LLC. In the porcine femoral bone, the remaining tissues from the bone were completely removed and washed with phosphate-buffered saline (PBS). The bone was pulverized into granules. The bone granules were then arranged in a tissue holder and kept in a SCCO_2_ vessel system (Helix SFE Version R3U, Applied Separations Inc., Allentown, PA, USA) with ml 60–75% ethanol as cosolvent. The SCCO_2_ instrument was then worked at 350 bar and 35–40 °C for 40–80 min to yield a bone graft.

### 2.2. Development of Acellular Dermal Matrix Powder

The porcine hide was trimmed off from the residual fat tissues and washed with phosphate-buffered saline (PBS). The skin was sliced to a thickness of about 0.7–1.0 mm, carefully rolled, and kept in a tissue holder, arranged inside a SCCO_2_ vessel system (Helix SFE Version R3U, Applied Separations Inc (Allentown, PA, USA), with a cosolvent of 10 mL 75% ethanol. The operation of the SCCO_2_ system was done at 200–350 bar and 30–50 °C for 40–80 min to produce an acellular dermal matrix sheet and subsequently washed in acetic acid (1–10%), hydrogen peroxide (10–35%), sodium hydroxide (0.1–1 N) and finally dried and freeze milled to make acellular dermal matrix powder [11].

### 2.3. Development of Putty from SCCO_2_-Derived Bone Graft Powder and Acellular Dermal Matrix Powder

The SCCO_2_-derived bone graft powder and acellular dermal matrix powder were used to prepare the bone putty. Bone graft powder size ranging from 0.25–2 mm, and the acellular dermal matrix powder size ranging from 0.1–0.25 mm were used for the putty formation. The ratio of the bone graft and acellular membrane powder varied from 10:1–4:1 to form the putty for the right consistency, mixed and prefilled in the syringe. Right before use, the putty is mixed with normal saline or blood so that the putty can be molded into desired shape and size.

### 2.4. Animal Efficacy Test—Rabbit Femoral Bone Defect Model

#### 2.4.1. Animals

New Zealand white rabbits weighing >3 kg (male) were used. All animals were purchased from the Animal Health Research Institute, Taipei, Taiwan. The animal’s earmarked and housed at 18~26 °C, humidity 30~75%, and one rabbit per cage, the experiments (Table 1) conducted were approved by the Institutional Animal Care and Use Committee (IACUC 19-228) of the National Defense Medical Center, Taipei, Taiwan.

#### 2.4.2. Surgical Procedure

New Zealand white rabbits were anesthetized by injecting Zolitel (Virbac, Taipei, Taiwan) 50, 15 mg/Kg BW; Xylazine (TCI, Tokyo, Japan), 7 mg/Kg BW, subcutaneously. The hair was shaved-off in both hind legs, cleaned with betadine, and then draped by aseptic surgical techniques. A 2.5 cm medial skin incision was made in the hind leg to use a medial approach to the distal femur. For performing a lateral arthrotomy of the stifle joint, the skin is retracted laterally. A defect of 5 mm in diameter and 10 mm in depth was created in the femur by using a surgical drill bit and a stopper set designed for creating critical-size defects. Both the right and left sides of the rabbit legs were operated on, with one defect in each leg. The drilling location was determined by the midpoint of the lateral condyle from the lateral fabella to the anterior portion of the lateral trochlea. The created defect was irrigated with normal saline. The diameter and the depth of the defect were measured by a sterilized ruler and a depth gauge to confirm the desired dimension. The measurement and the appearance of the defects were photographed to monitor the implanted sites. The defect sites were filled with the appropriate bone graft and putty. The bone graft and putty were prepared by mixing with normal saline. The endodermis and muscle tissues were stitched with absorbable sutures, and surface skins were done with non-absorbable Nylon sutures after bone graft and putty implantation. Antibiotic Penicillin was applied topically to the wound before suturing the wound. The wounds were covered with a swab of iodine to prevent inflammation or infection after stitching. The animals were administered postoperative Ketoprofen (3 mg/kg IM) analgesic twice for three consecutive days. No postoperative complications were noticed.

#### 2.4.3. Observations and Euthanasia

The conditions of the animals were monitored daily for the duration of the study. Appropriate action was taken for those animals with signs of postoperative complications, disease, pain, or stress. Moreover, care was conducted following current veterinary medical practice if an animal became injured and ill. Half of the experimental animals were assigned to sacrifice at 4 weeks, and the other half at 12 weeks were humanely euthanized according to the guidelines set forth by the AVMA Panel on Euthanasia [12]. Weights were measured and recorded immediately before sacrifice. The left and right femora of rabbits were collected. Tissues were placed in 4% formalin.

#### 2.4.4. X-ray and Micro-CT Analysis

After euthanasia, the bones were collected for micro-CT and histopathological investigation. All the bone specimen was scanned by an X-ray apparatus (Faxitron model MX20, Faxitron Bioptics, LLC., Tucson, AZ, USA) and scanned using a high-resolution microtomography scanner Quantum GX2 (PerkinElmer USA, Inc., Akron, OH, USA) to evaluate the regeneration of bone defect. The scanned images were reconstructed and analyzed using the SCANCO Evaluation Software (Scanco USA, Inc., Wayne, PA, USA). The measurement area of the critical size defect is a volume of interest in the field of view (FOV) 40 mm was selected from the reconstructed CT image stacks as the region of interest (ROI) to evaluate the radioopacity and quantified using Image J software (1.53f 25.)

#### 2.4.5. Histological Analysis

After harvesting, the bone samples were frozen, and sections were made for Masson trichrome and Alizarin S staining. For Masson trichrome and Alizarin S staining, we used the medullary level to evaluate bone collagen regeneration and calcium deposition relating to bone regeneration. Undecalcified histological specimens were embedded and immersed in carboxymethyl cellulose gel (SCEM, Japan) for 48 h. The frozen block was attached to the sample stage of the cryo-microtome (Leica CM3050 S; Illinois, USA) in the cryo-chamber. The coronal, vertical plane of the defect area was sectioned. 10 μm thick sections were cut and collected for Masson trichrome and Alizarin red S histomorphometric analysis.

#### 2.4.6. Hematoxylin and Eosin Staining

The remaining frozen samples were thawed rinsed in saline, fixed in 10% buffered formalin, and decalcified followed by paraffin embedding for routine H & E staining. Regarding the plane of the sections, the coronal, vertical plane of the defect area was sectioned. For H & E staining, we used the cortical level to evaluate the bone healing and regeneration of the defect. The stained bone sections were observed under a microscope (Olympus bx53, Texas, USA), and photographs were recorded at 100 micrometers magnification.

#### 2.4.7. Masson’s Trichrome Staining

Masson’s trichrome staining was conducted using a ready-to-use kit (Trichrome Stain (Masson) Kit, HT15, Sigma-Aldrich, St. Louis, MO, USA). Briefly, the tissue sections (thickness = 10 μm) were cut and placed on standard microscopy slides. Next, the sections were stained in Weigert’s hematoxylin for 5 min, washed with tap water for 5 min, and rinsed in distilled water. Then, the slides were stained in Biebrich scarlet-acid fuchsin for 5 min, rinsed in distilled water, incubated in phosphotungstic-phosphomolybdic acid for 5 min, dyed with aniline blue for 5 min, and fixed in 1% acetic acid for 2 min. Finally, the slides were rinsed in distilled water, dehydrated, and mounted. The measurement area of the critical size defect is an area of 2.5 × 2.5 × 3.14 (π) and expressed as mm^2^ was measured as the ROI. The photomicrographs were recorded at 200 micrometers and quantified using Image J software (1.53f 25.) National Institutes of Health, Bethesda, MD, USA.

#### 2.4.8. Alizarin Red S Staining

The alizarin red S solution was made in distilled water and adjusted to pH 4.1–4.3 with 10% ammonium hydroxide. Next, the sections were stained in alizarin red S solution for 5 min and then rinsed in distilled water for 5 min. Finally, the slides were dehydrated and mounted. The Masson’s trichrome and alizarin red S staining area of neogenic bone was measured by identifying the defect area and circle in each histological image of treatment groups. The neogenic bone area was derived by evaluating the percentage of new bone inside the area. The photomicrographs were recorded at 200 micrometers and quantified using Image J software (1.53f 25.) National Institutes of Health, Maryland, USA.

### 2.5. Statistical Methods

The data were analyzed using SPSS statistical software (SPSS, New York, NY, USA) and Microsoft Excel (Microsoft, Washington, DC, USA) and expressed as mean ± SD. Significant differences in measurement traits were analyzed using one-way analysis of variance (ANOVA) followed by least-significant difference (LSD) post hoc and Student’s *t*-test analysis. The significance was set at ns- (*p* > 0.05), *—(*p* < 0.05), **—(*p* < 0.001), ***—(*p*< 0.0001).

## 3. Results

### 3.1. Physical Comparison of Bone Graft and Putty

**Figure 1:**
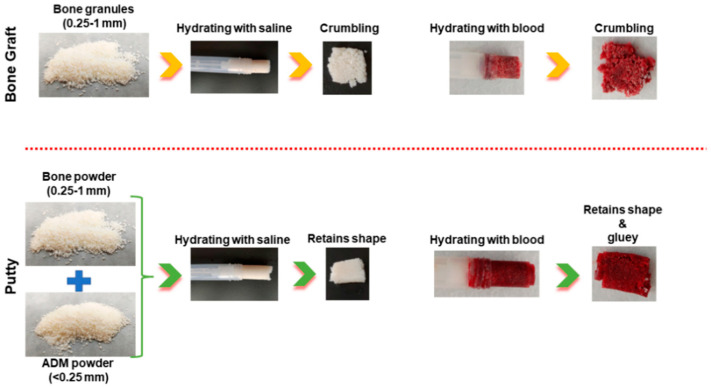
Schematic illustration of the physical nature of the bone graft and putty after hydrating with normal saline and blood.

The syringe-prefilled bone graft and putty were mixed well by pumping a few times and allowed to hydrate for 15 min with normal saline and blood. The bone graft and putty were squeezed out to evaluate the stickiness and cohesiveness. The bone graft was found to hold the tubular structure; however, the stickiness and cohesiveness were inadequate and found to be disintegrating and hard to retain the structure. The putty was found to retain the tubular structure. In addition, the putty depicted excellent stickiness and cohesiveness in both saline and blood medium (Figure 1).

**Figure 2:**
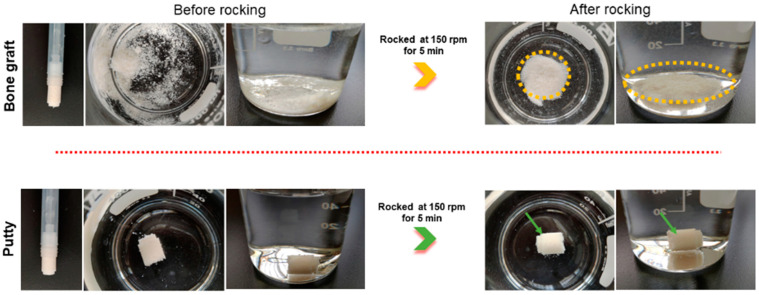
Schematic illustration of the hydrated bone graft and putty after rocking for five minutes in saline. The green arrow indicates cohesiveness.

To evaluate the stickiness and cohesiveness of the bone graft and putty in a physiological liquid medium. The bone graft and putty prefilled syringe were mixed with normal saline by pumping a few times and allowed 15 min to fully hydrate. The bone graft and putty were squeezed out, placed in a beaker with 100mL of normal saline, and rocked in a rocker for 5 min at 150 rpm. The bone graft crumbled immediately, dispersing the individual bone granules after placing it in the saline, even before rocking (circled in a yellow dotted line). The putty was found to retain the tubular structure after placing in the saline. After rocking in a rocker for 5 min at 150 rpm, the putty retained the tubular structure in the physiological saline medium, indicating that the putty can retain its shape and structure and attach to native bone (Figure 2).

### 3.2. Clinical Investigation: General Gross Observations

**Figure 3:**
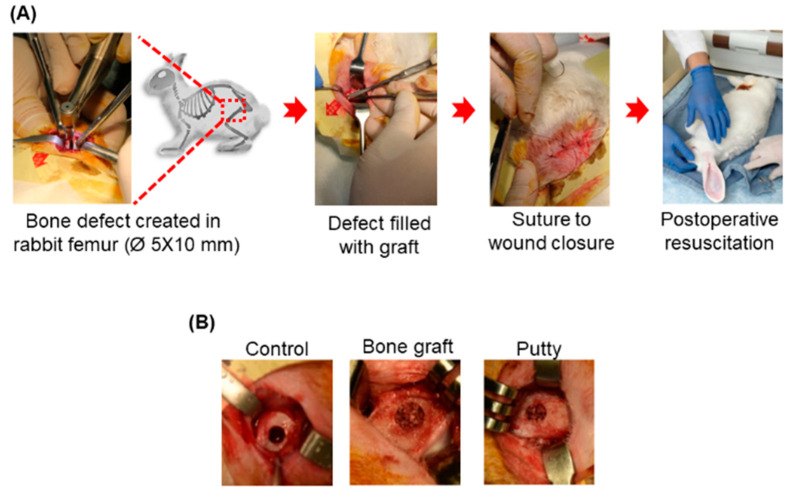
Schematic representative picture of the gross appearance of the critical-sized bone defects. (**A**) Creation of the critical-sized bone defect (Ø 5 × 10 mm) in rabbit femoral condyle. (**B**) Implanted critical-sized bone defect with bone graft and putty.

The critical-sized bone defects were established successfully in Figure 3. The surgeries were uneventful, and all animals recovered without incident. The bone graft and putty were implanted into the defect of the femur bone. All the experimental animals survived the lesion creation surgery without postoperative complications, and the suture wound healed without complications within 7–10 days after surgery. The experimental animal’s diets and behavior were normal post-operatively, like standup and walking on the first day after the surgery and carrying out their normal daily activities, such as feeding and drinking, within 48 h after surgery. The surgical wounds were closed and healed, and there was no sign of infection at the final evaluation point. The experimental animals showed no clinical problems post-operatively.

### 3.3. Radiographic Evaluation of Bone Graft and Putty-Implanted Animals

**Figure 4:**
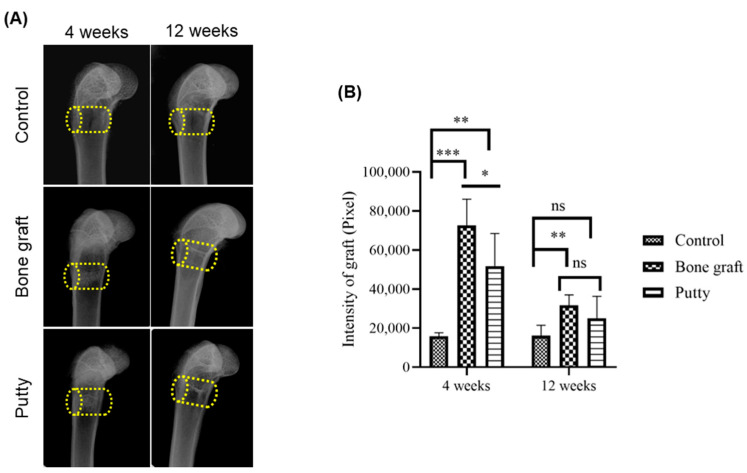
Lateromedial radiographic assessment of the critical-sized bone defects. (**A**) Representative lateromedial radiographic image. (**B**) Radiographic quantification of resorption of the bone graft and putty after implantation. The yellow cylinder indicates the defect site. The significance was set at ns- (*p* > 0.05), *—(*p* < 0.05), **—(*p* < 0.001), ***—(*p*< 0.0001).

The latero-medial radiographs depicted defined radiolucent bone defects in the femora of the experimental groups (Figure 4A). At four weeks, bone graft and putty implanted defects depicted increased radiopacity in the femora due to their increased radiographic density than the adjacent bone and relatively higher than the control group (Figure 4B). A significant (*p* < 0.0001) increase in the radiographic density was found in the bone graft implanted defect compared to the control at four weeks. Putty implanted defect at four weeks showed a significant (*p* < 0.001) increased radiographic density relative to control bone defect. The bone graft implanted defect at four weeks showed a significant (*p* < 0.01) increased radiographic density compared to the putty implanted defect.

At 12 weeks, bone graft implanted defects depicted significant (*p* < 0.001) increased radio-opacity in the femora relative to the control group (Figure 4B). However, the putty implanted defect showed a non-significant increase in the radiographic density relative to the bone graft and control group.

### 3.4. Role of Putty on Osteogenesis by Micro-CT Examination

**Figure 5:**
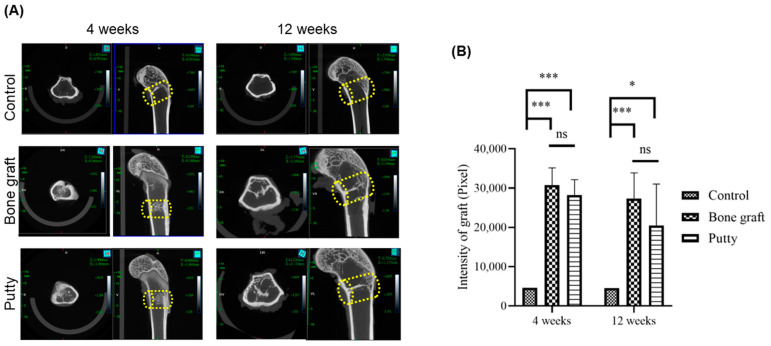
Computed tomography (µ-CT) assessment of the critical-sized bone defects. (**A**) Representative transverse and coronal CT images. (**B**) µ-CT signal quantification of resorption of the bone graft and putty after implantation. The yellow cylinder indicates the defect site. The significance was set at ns- (*p* > 0.05), *—(*p* < 0.05), ***—(*p*< 0.0001).

Micro-CT examination offers an exclusive understanding of the radiographic signal and performance of the bone graft and putty in bone regeneration. At 4 and 12 weeks, in the control group, bone defects can be distinguished in the coronal and transverse planes (Figure 5A,B). However, bone graft and putty implanted defects looked partly filled and partially degraded implanted materials, indicating resorption demonstrating new bone regeneration in the region of interest (ROI) in the cortical area. At four weeks, the bone graft and putty implanted defects depicted significant (*p* < 0.0001) radiographic signals for partial filling and osteogenesis compared to the control group. However, no significant difference was observed in the radiographic signal between the bone graft and putty-implanted defects.

At 12 weeks, bone graft (*p* < 0.0001) and putty (*p* < 0.001) implanted defects depicted significantly increased radiographic signal in the femora relative to the control group (Figure 5B). However, the putty implanted defect showed no significant changes in the radiographic signal relative to the bone graft group.

### 3.5. Role of Putty on Osteogenesis by Histological Assessment

Femoral bone defect histological examinations were performed postoperatively at 4 and 12 weeks to evaluate the osteogenic and regenerative potential of bone graft and putty that elicit bone formation in the critical-sized bone defect.

**Figure 6:**
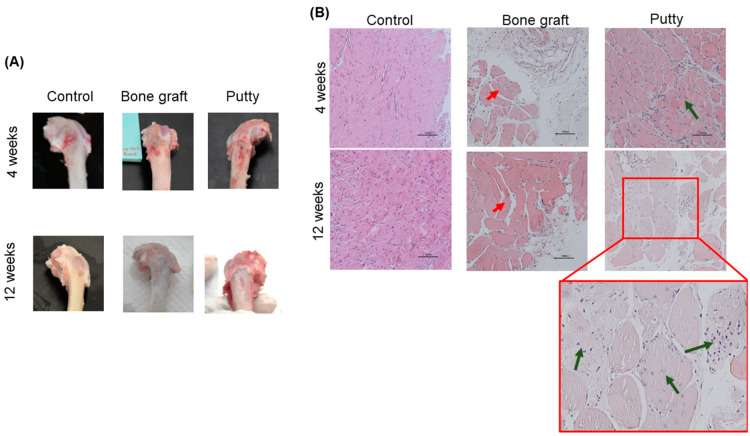
Macroscopic and microscopic assessment of critical-sized bone defect repair in rabbit femurs. (**A**) Representative gross images of the femoral condyle of the critical-sized bone defect. (**B**) Representative femoral sections were stained with Hematoxylin and of the critical-sized bone defect (Scale bar: 200 µm). The red arrow indicates bone granules. Green indicates bone granules with new bone formations.

At four weeks, the control group showed the defect was filled with moderate fibrous tissue and elevated infiltrating cells. The bone graft implanted defects are depicted with dispersed bone graft granules divided by slight osteoid tissue, few osteogenic cells, and surrounded by spongy bone trabeculae (Figure 6B). The putty implanted defects showed complete filling with bone graft granules divided by collagen fibers, osteoid tissue, osteogenic cells, osteoblasts, osteoclasts, and a few fibroblasts surrounded by spongy bone trabeculae.

At 12 weeks, the control group showed the defect is occupied completely with fibrous tissue and moderate infiltrating cells without bone repair. The bone graft implanted defects are depicted with a few dispersed bone graft granules divided by osteoid tissue and osteogenic cells and surrounded by spongy bone trabeculae (Figure 6B). The putty implanted defects showed complete filling with bone graft granules divided by collagen fibers, osteoid tissue, and newly formed bone trabeculae centrally and connected to the peripheral newly formed lamellar bone.

### 3.6. Role of Putty on Osteogenesis by Masson Trichrome Staining in the Bone Defect Area

Masson trichrome staining was done in the areas of interest to examine the potential histological alterations related to the newly formed collagen in the new bone formation within the regenerated bone defect areas.

**Figure 7:**
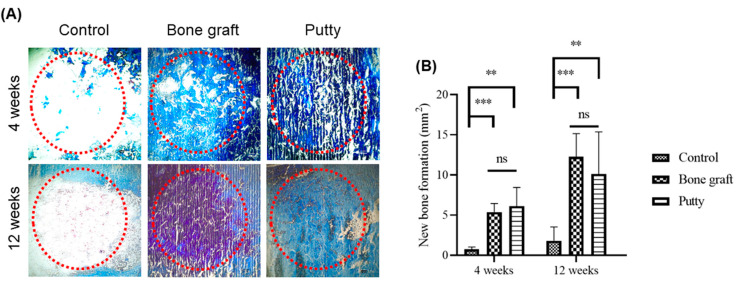
Masson trichrome staining histochemical examination of new bone collagen in the critical-sized bone defects. (**A**) Representative femoral sections were stained with Masson trichrome staining of the critical-sized bone defect. (**B**) Masson trichrome staining quantification of new bone formation in the bone graft and putty after implantation. The red dotted circle indicates the critical-sized bone defect. The significance was set at ns- (*p* > 0.05), **—(*p* < 0.001), ***—(*p*< 0.0001).

At four weeks, bone graft and putty implanted defects depicted increased Masson trichrome staining relatively higher than the control group (Figure 7A,B). A significant (*p* < 0.0001) increase in the Masson trichrome staining was found in the bone graft implanted defect compared to the control at four weeks. Putty implanted defect at four weeks showed a significant (*p* < 0.001) increased Masson trichrome staining relative to control bone defect, indicating osteogenesis leading to the new bone formation by putty in the early stages of bone regeneration. However, no significant alterations in Masson trichrome staining bone graft implanted defect compared to putty implanted defect.

At 12 weeks, bone graft implanted defects a significant (*p* < 0.0001) increase in the Masson trichrome staining was found in the bone graft implanted defect compared to the control. Putty implanted defect at 12 weeks showed a significant (*p* < 0.001) increased Masson trichrome staining relative to control bone defect. However, no significant changes in Masson trichrome staining bone graft implanted defect relative to putty implanted defect.

### 3.7. Role of Putty on Osteogenesis by Alizarin Red S Staining in the Bone Defect Area

Alizarin red is generally employed to stain and recognize calcium-containing osteocytes in differentiated cells from mesenchymal stem cells, leading to new bone formation in the bone defect areas.

**Figure 8:**
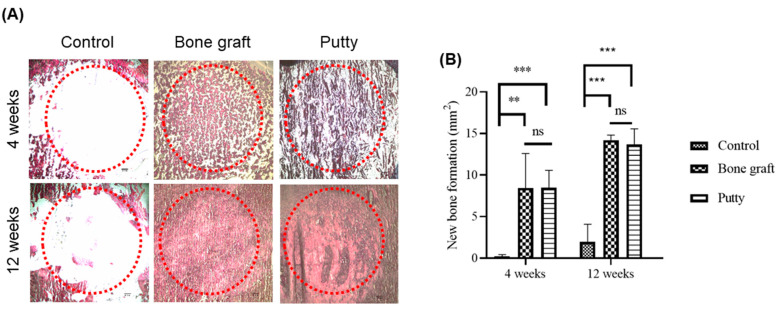
Alizarin Red S staining histochemical examination of new bone collagen in the critical-sized bone defects. (**A**) Representative femoral sections were stained with Alizarin Red S staining of the critical-sized bone defect. (**B**) Alizarin Red S staining quantification of new bone formation in the bone graft and putty after implantation. The red dotted circle indicates the critical-sized bone defect. The significance was set at ns- (*p* > 0.05), **—(*p* < 0.001), ***—(*p*< 0.0001).

At four weeks, bone graft and putty implanted defects depicted increased Alizarin Red S staining relatively higher than the control group (Figure 8A,B). Alizarin Red S staining significantly (*p* < 0.001) increased in the bone graft implanted defect compared to the control at four weeks. Putty implanted defect depicted a significant (*p* < 0.0001) increase in Alizarin Red S staining compared to control bone defect, indicating osteogenesis leading to the new bone formation by putty in the early stages of bone regeneration. The bone graft implanted defect compared to the putty implanted defect depicted non-significant changes in Alizarin Red S staining.

At 12 weeks, bone graft implanted defects a significant (*p* < 0.0001) increase in the Alizarin Red S staining was found in the bone graft implanted defect compared to the control. Putty implanted defect at 12 weeks showed a significant (*p* < 0.0001) increased Alizarin Red S staining relative to control bone defect, indicating osteogenesis leading to the new bone formation was complete by putty during bone regeneration. The bone graft implanted defect compared to the putty implanted defect portrayed non-significant changes in Alizarin Red S staining.

## 4. Discussion

To the best of our knowledge, this is the first study to evaluate the “putty” made solely by employing supercritical fluid extraction technology from porcine bone and hide; the putty is easy to handle and efficient in bone regeneration. The critical-sized bone defect management is considered a big task, and substitute materials for implantation are required [13]. At present various approaches have been established for the augmentation of critical-sized bone defects, which include autologous bone transplantation and the use of allografts [14,15]. Nevertheless, the aforementioned strategies had limitations that hindered their frequent use [16]. Bone graft alternatives offer physicians an “off the shelf” substitute to help heal defects. The perfect bone graft material criteria are; handling well during surgery, effortlessly implanted into the defect, and finally functioning as an osteoconductive scaffold to assist and restore the defect. The vast number and numerous variations of bone graft materials that have been established as well as used clinically are noteworthy [17,18], indicating that we still need to find the ideal bone graft candidate for clinical use [17]. In the present study, the putty retained the tubular structure after placing it in the saline, indicating that it can retain its shape and structure and attach to bone. The putty produced had excellent physical properties, such as retaining the structure and stickiness, along with excellent bone regenerative properties (Table 2).

The challenging issue with non-healed critical-sized bone defects in humans is that the defects do not tend to restore spontaneously without supplementary intervention [8,19]. Recently, bone tissue engineering has been used to fasten the osteo-regeneration process [19,20,21]. The perfect artificial bone grafting material should be cost-effective, easily used, play a role in inducing the osteoconductive and osteoinductive extracellular matrix microenvironment, and has outstanding mechanical properties. Furthermore, the bone graft should be biocompatible and competent to assist cell attachment and growth [19,22]. Bone graft alternatives made with the main ingredients of the unique composite, type I collagen and calcium phosphate, is a reasonable choice. Biomaterials created using collagen were used for a long time in various clinical applications before bone graft materials were used [17,23]. The sophistication and multifaceted nature of the type I collagen molecule have led to numerous challenges in processing of biomaterials related to antigenicity and immunogenicity [17]. In the present study, we used SCCO_2_ produced bone graft that contains a native collagen scaffold with type I collagen with proven biocompatibility, osteoconductive, and osteoinductive with outstanding mechanical properties. The most vital property is without immune-related adverse rejections, which enhances bone healing and regeneration in the critical defect in the rabbit model.

Demineralized bone matrix (DBM) is an acid-extracted demineralized allograft compound containing collagens, noncollagenous proteins, and growth factors. Subsequent decalcification, growth factors can be free from the surrounding mineral components and completely use their osteoinductive nature, and the residual collagen offers a 3D configuration for osteoconduction [2]. Depending on the various production protocol, DBM is accessible in diverse types such as sponges, freeze-dried powder, gel, paste, putty, and strips. Between these, DBM powder owns a huge surface area for revealing collagen at the graft site, using good osteoinductive capacity [24]. Nevertheless, the absence of mechanical strength and constancy, as well as problems in handling, are the chief downsides of DBM powder for clinical use [2,25]. In the current investigation, the putty implanted defect showed increased radiographic density relative to the control bone defect. Meanwhile, bone graft and putty implanted defect showed increased radiographic density compared to the control defect, indicating osteogenesis and osteointegration of bone graft and putty in the early stages of bone regeneration. We used SCCO_2_ produced bone graft that holds residual collagen and offers a 3D configuration with type I collagen for osteoconduction with reputable biocompatibility and excellent mechanical strength, which augments bone healing and regeneration in the critical defect in the rabbit model.

The addition of collagen to bone graft boosted clinical handling and important function in particle resorption mechanisms [26,27]. The precise effect of collagen on bone tissue as grafting material encouraged biomaterial resorption and functioned substantially in the osteoconductive properties [28]. In the bone remodeling process, the possible role of collagen is on the newly recruited osteoblast lineage cells positioning directly beside osteoclasts displaying endocytic collagen receptors involved in collagen internalization and cell migration [27,29]. The collagenated porcine bone displayed elevated osteoconductive properties and is generally resorbed, subsequently leading to an augmented percentage of newly formed bone and a simultaneous decrease in residual grafting material [27,28]. In the present investigation, we used a SCCO_2_-produced bone graft mixed with ADM powders to produce putty that made it easy for clinical handling and encouraged biomaterial resorption leading to a bone remodeling process, which increases bone healing and regeneration in the critical defect.

In the present study, the bone graft implanted defects are shown with a few dispersed bone graft granules divided by osteoid tissue and osteogenic cells surrounded by spongy bone trabeculae. The putty implanted defects depicted the complete filling of the defect with bone graft granules divided by collagen fibers, osteoid tissue, and newly formed bone centrally and connected to the newly formed bone. We used bone graft, putty made with bone graft, and ADM to evaluate the critical bone defect model’s regenerative potential and osteoinductive nature. The outstanding bone regeneration and new bone formation in the segmental defect model indicate that bone grafts produced using the SCCO_2_ technique improved the regenerative ability of bone grafts [16]. The bone graft produced using the SCCO_2_ technique possessed the native pore structure of porcine bone with high porosity. The porosity and pore size function as a dynamic role in the bone scaffold’s cell seeding, diffusion, and mechanical strength. Bone remodeling was accomplished by an enormously porous bone scaffold that aids vascularization, osseointegration, osteoblast, and osteoclast infiltration. Decreased bone porosity limits cell growth and infiltration for bone regeneration [16,30]. The porous nature of the bone graft was known to play a vital role in cell adhesion and proliferation, subsequently leading to bone regeneration.

In the present study, the bone graft and putty implanted defect depicted an increase in Masson trichrome, indicating bone collagen regeneration, and Alizarin Red S staining indicating calcium deposition for bone regeneration compared to control bone defect, demonstrating osteogenesis leading to the new bone formation during bone healing and regeneration. However, the X-ray and micro-CT demonstrated the bone graft and putty resorption and integration of the new bone to host bone in the implanted defect leading to the reduction of radiographic intensity reported at 12 weeks. Our bone graft is an excellent bone substitute, an absorbable calcium salt established to regenerate the bone defect, suggesting the bone graft and putty have good potential for osteoconductivity and promoting new bone formation. The microporosity of the bone graft is well-preserved by SCCO_2_ manufacturing. In addition, the SCCO_2_ process preserved the native structure of the bone graft; it also conserved the diverse range and network of pores from micro- to nanosize, which are necessary for angiogenesis and encourage both bone growth and reorganization [10,31]. However, the bone grafts in the market are mass-produced by high-temperature sintering, which modifies the native pores in the bone, impeding bone regeneration [10,16]. The bone graft contains a high Ca/*p* ratio of 1.75, indicating that the chemical composition of the bone graft is good. In addition, high content of Ca and *p* induces osteogenesis [10,16]. The bone grafts’ chemical contents and structure were found to be similar to that of human grafts. Additionally, bone grafts showed excellent in vitro and in vivo biocompatibility and promising new bone formation [10,16,32].

The statistical comparison within the bone graft and putty implanted group between 4 and 12 weeks was performed and reported for radiologic and histomorphometry evaluations. In that radiologic data, X-rays in bone graft implanted defect show a significant (*p* < 0.001) decrease in radiographic density at 12 weeks compared to 4 weeks. In putty implanted defects show a significant (*p* < 0.0001) decrease in radiographic density at 12 weeks compared to 4 weeks, indicating bone resorption and osteointegration into the host bone. However, the micro-CT data showed no alterations in the radiographic density between 4 and 12 weeks; this might be because Micro-CT scanners capture a series of 2D planar X-ray images and reconstruct the data into 2D cross-sectional slices. These slices can be further processed into 3D models for analysis. Therefore, the implanted bone graft and putty have complex structures, including the pores in the bone granules, cracks, and the structure of the acellular dermal matrix in the putty will be considered in the 3D physical objects for analysis. This may be the reason for no alteration in the radiographic density between 4 and 12 weeks. However, compared with the control defect, a significant increase in the radiographic density was observed in both bone graft and putty as well as 4 and 12 weeks (refer to supplementary data Figures S1 and S2).

Masson trichrome and Alizarin S staining’s statistical comparison was performed within the same group between 4 and 12 weeks. Masson trichrome staining in bone graft implanted defect shows a significant (*p* < 0.0001) increase at 12 weeks compared to 4 weeks. In putty implanted defects show a significant (*p* < 0.001) increase at 12 weeks compared to 4 weeks, indicating bone collagen regeneration. Alizarin S staining in bone graft implanted defect shows a significant (*p* < 0.001) increase at 12 weeks compared to 4 weeks. In putty implanted defects show a significant (*p* < 0.0001) increase at 12 weeks compared to 4 weeks, indicating calcium deposition (refer to supplementary data Figures S3 and S4). Therefore, bone collagen regeneration and calcium deposition subsequently enhance bone regeneration.

Acellular dermal matrices (ADM) are used clinically for diverse purposes, including treatment of reconstructive surgery and chronic wound repair. ADM preserves the original extracellular matrix scaffold comparable to the biomechanical properties of the skin and does not elicit an immune response. It also supports the speedy and directed proliferation of infiltrated cells and enables revascularization and constancy during the repair process [33]. The ADM structure is comparable to a human acellular dermal matrix [34]. In addition, the ADM contained interwoven porous collagen fibers. The chemical content of ADM showed an abundance of protein, with the absence of carbohydrates and fat [35].

The SCCO_2_-produced ADM showed the native collagen scaffold with its normal porosity, and the structural pattern of collagen fiber remains intact. Porosity plays an essential role in cell seeding, diffusion, and mechanical strength efficiency. Porosity permits the cells to distribute homogeneously and sustain the effective transport of nutrients, oxygen, growth factors, and waste products. In addition, the ADM scaffold owns reasonable stiffness to tolerate the adjacent mechanical stresses throughout tissue neogenesis. The ADM scaffold also offers a biochemical and physical environment comparable to native tissue, which is vital in promoting cell adhesion, proliferation, differentiation, and tissue neogenesis [35,36]. The ADMs extracellular matrix (ECM) contains mainly collagen, structural proteins, and a basement membrane. Collagen offers excellent properties of elasticity, tensile strength, and compressibility [37]. The ADM protects the wound and attenuates inflammation, permits the migration of cells for repair, and elicits epithelization, thereby enhancing wound healing [35,38]. In the present investigation, we used a SCCO_2_-produced bone graft mixed with ADM powders to produce putty, which contains a native collagen scaffold with its normal porosity that permits the cells to distribute homogeneously and sustain the effective transport of nutrients, oxygen, and growth factors, in turn, promotes cell adhesion, proliferation, differentiation, and tissue neogenesis that enhances bone healing and regeneration in the critical defect.

## 5. Conclusions

In the present study, the SCCO_2_-produced bone graft and putty were formulated by combining bone graft with ADM powder to treat the critical defect in the rabbit model. The putty produced had excellent physical properties, such as retaining structure and stickiness. In both putty and bone, graft-filled defects between 4 and 12 weeks, an increase of bone has been observed. The possible mechanism of improved healing and regeneration of critical defects by bone graft and putty might be the porosity of ADM that permits the cells to distribute homogeneously and sustain the effective transport of nutrients, oxygen, and growth factors, which in turn, promotes cell adhesion, proliferation, differentiation, and tissue neogenesis. The radiographic imaging, histology, Masson trichrome, and Alizarin Red S staining confirm the healing and regeneration of critical defects by bone graft and putty. In addition, putty makes it easy for clinical handling and contains type I collagen, encouraging biomaterial resorption leading to a bone remodeling process, osteoconductive and osteoinductive, which increases bone healing and regeneration in the critical defect.

## Figures and Tables

**Table 1:** Animal groups and implantation details in rabbit femoral bone defect model.

Groups	Implantation	Number of Animals
Control	None	8
Bone graft	Bone graft	12
Putty	Putty	12

**Table 2:** Comparison of bone graft and putty.

Properties	Bone Graft	Putty
Composition	Bone powder	Bone powder and ADM powder
Physical form	Powder	Putty
Chemical nature	Natural bone minerals and collagen	Natural bone minerals and collagen + natural dermal collagen
Pliability	Not applicable	Versatile
Usage	Needs surgical tools	Ready to use after rehydration
Stability/ retaining nature in fluid	Easily crumbles	Retains the shape and structure
Adhesiveness	Low	Relatively good
Osseointegration	Good	Good

## Supplementary Materials

The following supporting information can be downloaded at: https://www.mdpi.com/article/10.3390/biomedicines10112802/s1, Figure S1: Radiographic quantification of resorption of the bone graft and putty after implantation; Figure S2: μ-CT signal quantification of resorption of the bone graft and putty after implantation; Figure S3: Masson trichrome staining quantification of new bone formation in the bone graft and putty after implantation; Figure S4: Alizarin Red S staining quantification of new bone formation in the bone graft and putty after implantation.
