# Supplementary material for: Regenerative Efficacy of Supercritical Carbon Dioxide-Derived Bone Graft Putty in Rabbit Bone Defect Model
Source: Biomedicines. 2022 Nov 3;10(11):2802. doi: 10.3390/biomedicines10112802 (PMC9687147; doi:10.3390/biomedicines10112802)

Supplementary Figure S1

Radiographic quantification of resorption of the bone graft and putty after implantation

Comparative analysis: 4 weeks Vs 12 weeks

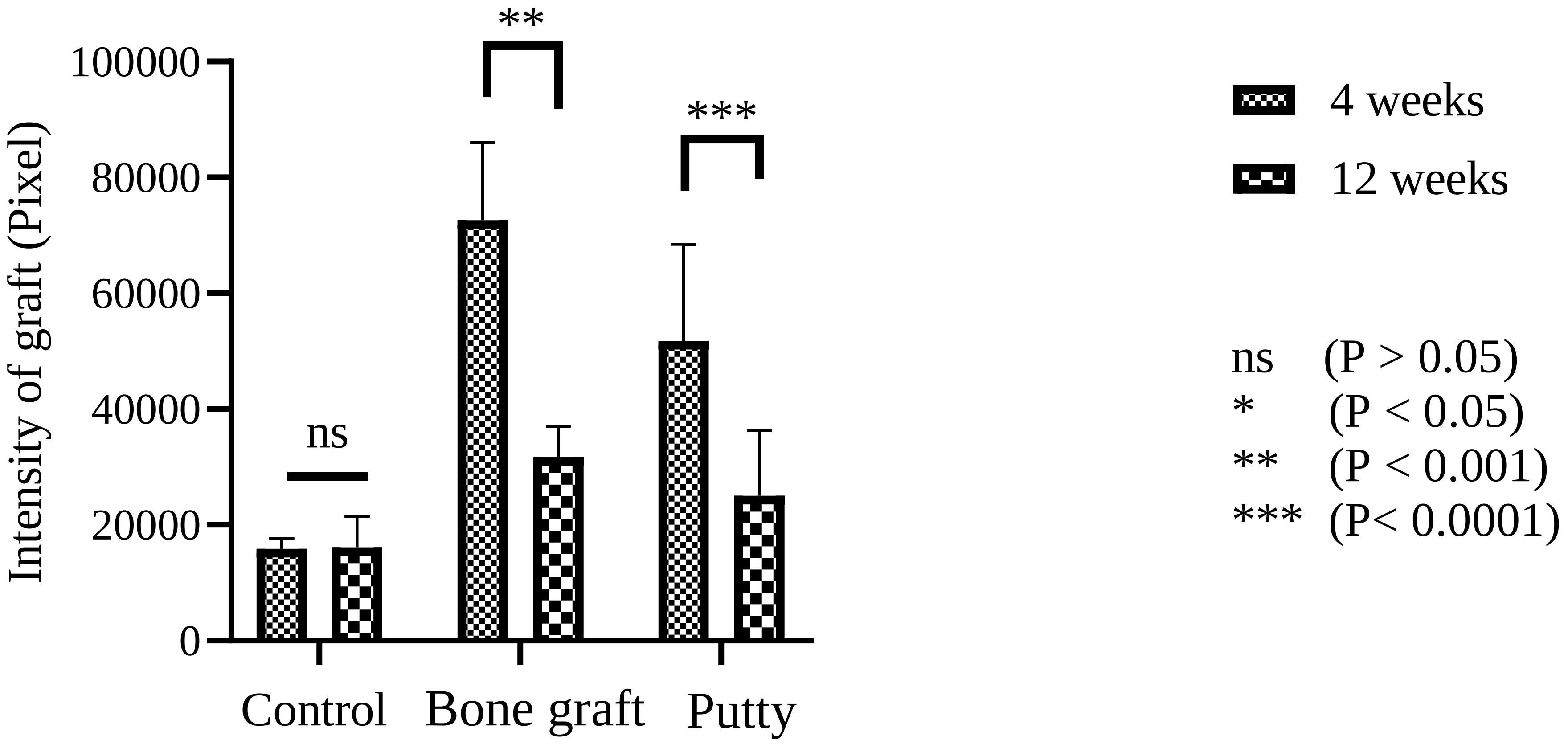

Supplementary Figure S2

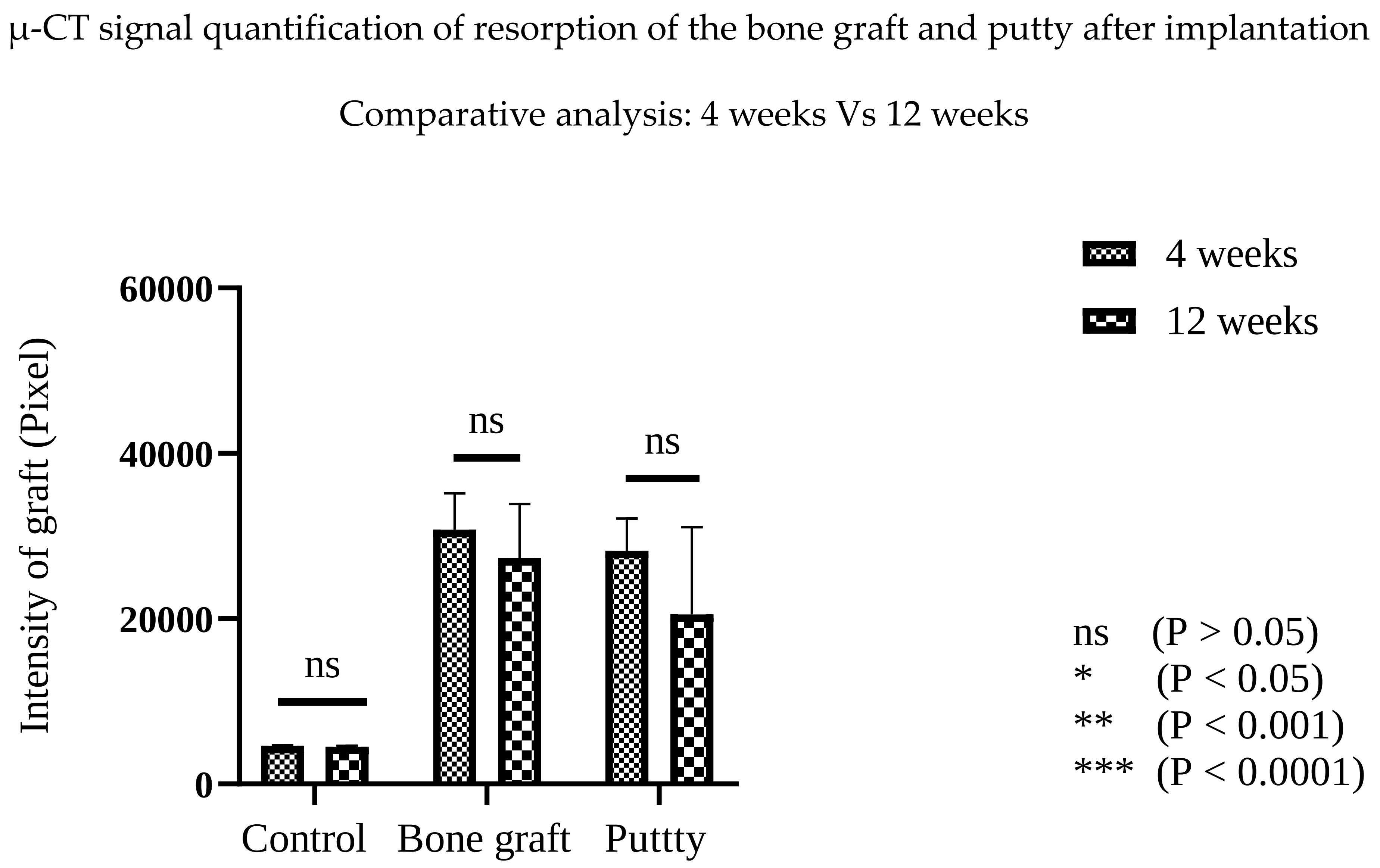

Supplementary Figure S3

Masson trichrome staining quantification of new bone formation in the bone graft and putty after implantation.

Comparative analysis: 4 weeks Vs 12 weeks

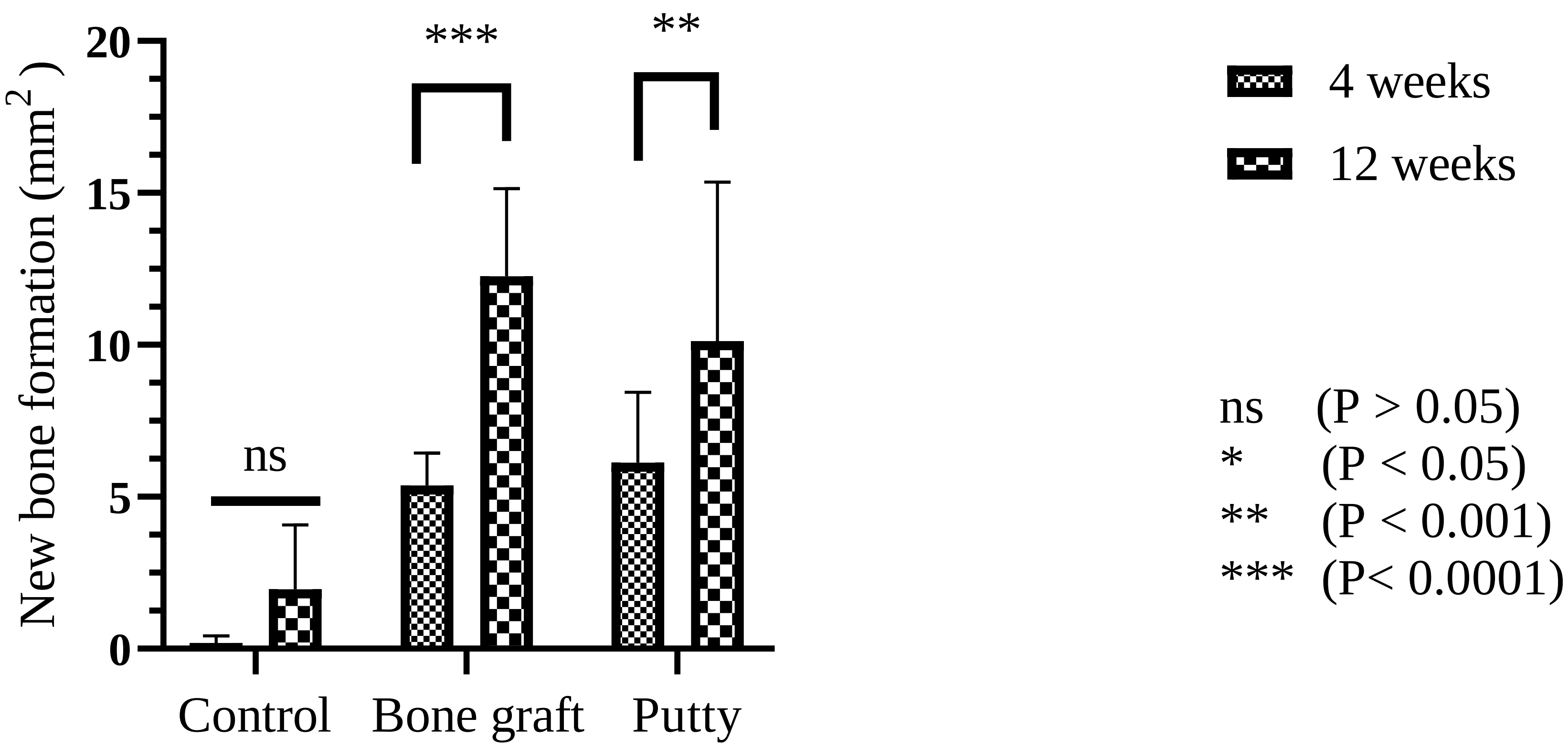

ns (P > 0.05)
* (P < 0.05)
** (P < 0.001)
*** (P< 0.0001)

Supplementary Figure S4

Alizarin Red S staining quantification of new bone formation in the bone graft and putty after implantation

Comparative analysis: 4 weeks Vs 12 weeks

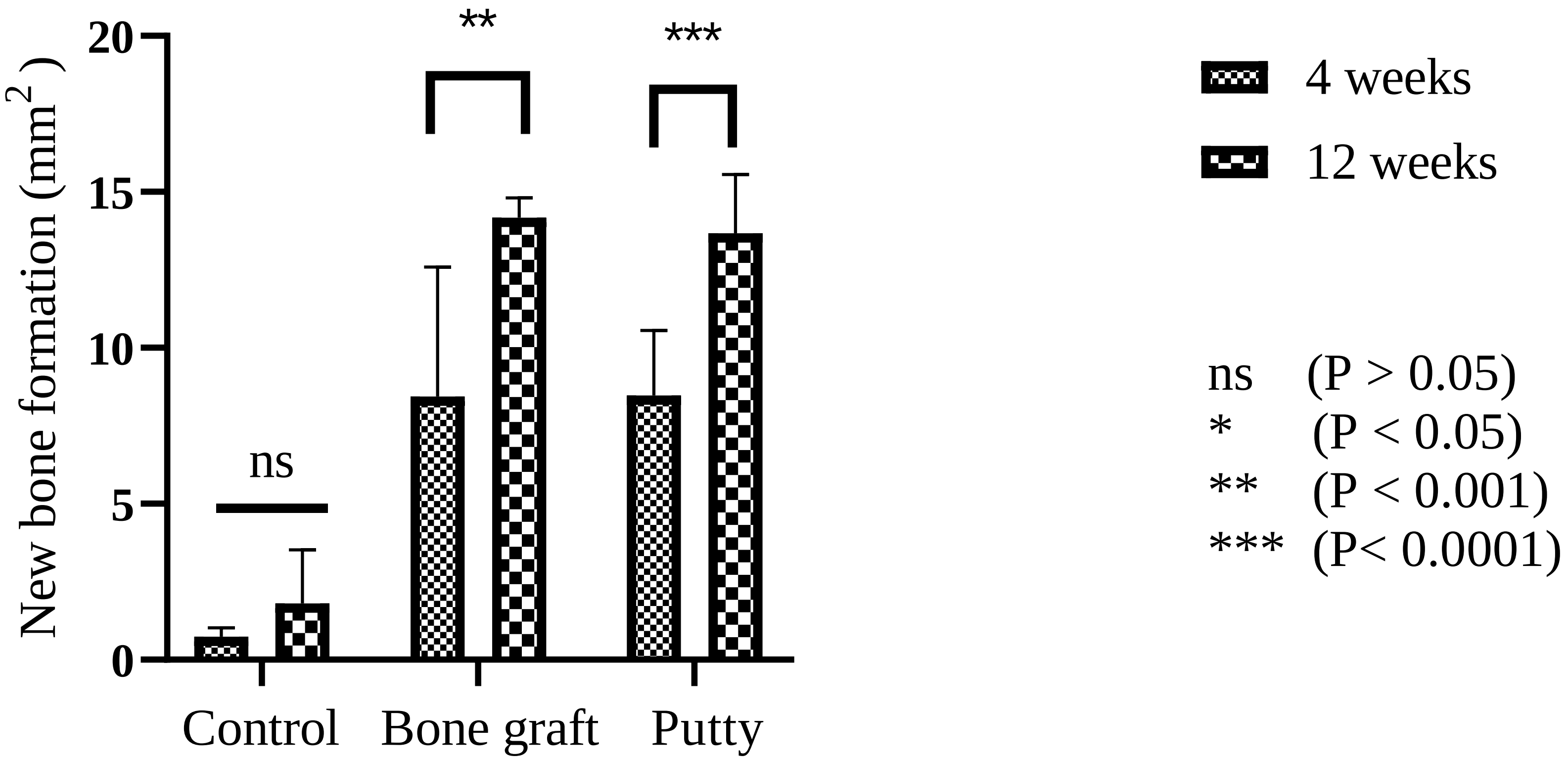

Supplement: Supplementary file 1 [file biomedicines-10-02802-s001.zip › biomedicines-1973059-supplementary.pdf]
